# Determinants of Caesarean Section Delivery in the Southern Region of India: Insights From the National Family Health Survey 5

**DOI:** 10.7759/cureus.83698

**Published:** 2025-05-08

**Authors:** Singh Jigyasa, Jagriti Annu, Tej Bali Singh

**Affiliations:** 1 Department of Obstetrics and Gynaecology, Institute of Medical Sciences, Banaras Hindu University, Varanasi, IND; 2 Centre of Biostatistics, Institute of Medical Sciences, Banaras Hindu University, Varanasi, IND

**Keywords:** cesarean delivery (cd), cesarean section rate, maternal child health, maternal mortality, normal vaginal delivery, pregnancy complications

## Abstract

Introduction

Global caesarean section (CS) rates have increased substantially during the last 20 years. High prevalence of CS delivery has been seen in the southern region of India. The objectives of this study are to determine the prevalence of delivery by CS in the southern region of India and to identify the significant factors associated with delivery by CS.

Methods

For this study, the data from the fifth round of the National Family Health Survey (NFHS-5) related to the southern region of India are considered. Descriptive statistics, Pearson’s chi-square test, and multiple logistic regression analysis were used.

Results

The percentage of CS delivery in the southern region of India was 46%, which exceeded the guidelines of the World Health Organisation of a 10-15% threshold. Women belonging to the age group of 35-49, living in urban areas, were more likely to deliver by CS as compared to the women of the age group of 15-24 and in rural areas. Muslim, low-wealth quintile, and illiterate women were less likely to deliver by CS. Multiple logistic regression analysis revealed that women aged 25-34 years, those with higher education, and those from wealthier households, birth order of one or two, late age at first birth (≥30 years), overweight or obesity, and more antenatal care (ANC) visits were also significantly associated with delivery by CS. Deliveries in private facilities had three times higher odds of CS (aOR: 3.00; 95% CI: 2.82-3.19).

Conclusion

This study highlights the high prevalence of CS deliveries in the southern region of India. It underscores the urgent need for government interventions to reduce unnecessary CS and to promote safe, evidence-based, and natural childbirth practices.

## Introduction

Globally, caesarean section (CS) rates have surged from approximately 7% in 1990 to 21% in recent years, with projections indicating a continued upward trajectory throughout the current decade [1]. In obstetrics and human culture, CS are used when vaginal delivery is either impractical or presents too many dangers for the mother or the baby or both [2]. When medically indicated, CS can save a mother's life and the life of her infant [3].

If CS is not required, then they can lead to a variety of negative short- and long-term health consequences for both mothers and newborns, including infection in the mother, uterine haemorrhage, respiratory distress in the infant, and hypoglycemia [4]. Compared to vaginal births, women who have previously had a caesarean delivery and give birth via CS are more likely to not continue breastfeeding [5]. Longer hospital stays and higher out-of-pocket costs are associated with CS deliveries [6].

Many factors have been discovered to influence the rates of CS in India and around the world. These range from individual characteristics of the mother, child, and community level. Characteristics of mothers associated with CS are age at marriage [7], women’s age at delivery [8], and education level [9]. Characteristics of a baby associated with CS are birth order and size of the child at birth [10]. Demographic and community-level factors that affect CS are caste [11], place of residence [10], and wealth quintile [12].

A number of studies have focused on the rising percentage of CS births worldwide and in India specifically [13]. It is particularly alarming that the prevalence of CS deliveries in private facilities is generally significantly greater than in public hospitals [14]. There is a high geographical variability of CS within India, as well as prevalence ranging from 5.2% in Nagaland to 60.7% in Telangana [15]. High prevalence of CS delivery has been seen in South India. CS rates in Telangana, Kerala, Andhra Pradesh, and Karnataka are 60.7%, 42.4%, 42.4%, and 31.5%, respectively. At least one in four women in South India gives birth via CS [16]. Understanding the factors associated with CS deliveries is crucial for informing public health interventions and improving maternal and child health outcomes.

The objectives of this study are to determine the prevalence of delivery by CS in the southern region of India and to identify the significant factors associated with delivery by CS.

## Materials and methods

Source of data

The data used in this study were obtained from the Demographic and Health Surveys (DHS) Program’s official website after obtaining necessary permission. The National Family Health Survey round 5 (NFHS-5) datasets are publicly available for academic use upon registration and approval through a formal request process. NFHS-5 is a cross-sectional study, conducted during 2019-2021 among 29 states and the seven union territories. Since 1992-1993 (NFHS 1), it has been done under the direction of the Ministry of Health and Family Welfare. The International Institute for Population Sciences (IIPS), Mumbai, serves as the nodal agency for the planning, implementation, data analysis, and report writing of the survey. NFHS-5 collected comprehensive information on fertility, family planning, neonatal and child morbidity and mortality, maternal and reproductive health, and the nutritional status of women and children, among other topics. A two-stage sampling design was employed for rural areas and a three-stage design for urban areas. A total of 724,115 women were successfully interviewed, yielding a response rate of 97%.

Sample size

This study utilized data from the NFHS-5 dataset (2019-2021), which surveyed a total of 724,115 women across India. From this, 110,470 women were identified as residing in the southern region of India, which includes the states of Andhra Pradesh, Karnataka, Kerala, Tamil Nadu, Telangana, and the Union Territories of Lakshadweep and Puducherry. Among them, a final analytical sample of 22,365 women, those in the reproductive age group (15-49 years) who had a live birth in the five years preceding the survey, were included in this study. However, due to missing responses, the sample size varied slightly for certain variables: 22,311 for the size of the child at birth; 21,477 for the respondent’s body mass index (BMI); and 3,104 for the respondent’s working status.

Outcome variable

Delivery by CS (DCS) is the outcome variable, which was evaluated as a binary outcome (yes or no).

Independent variables

The study included several independent variables, such as respondent’s age group (15-24, 25-34, and 35-49), respondent’s place of residence (urban and rural), respondent’s religion (Hindu, Muslim, and others), respondent’s working status (Yes and No), respondent’s educational level (no education, primary, secondary, and higher), wealth quintile of the respondent (poorest, poorer, middle, richer, and richest), age at first birth (years) of the respondent (1, 2, 3, and more than 3), birth order of child (1, 2, 3, and more than 3), the respondent’ BMI (underweight, normal weight, overweight, and obese), number if antenatal care (ANC) visits (none, less than four, and at least four), size of child at birth (large, average, and small), place of delivery (private and government), history of terminated pregnancy of the respondent (yes and no), and gender of child (male and female).

Statistical analysis

We first conducted univariate analysis to describe the background characteristics of the respondents using frequencies and percentages. This was followed by bivariate analysis, using Pearson’s chi-square test, to examine associations between CS delivery and each independent variable. Subsequently, multiple logistic regression analysis was performed to identify the adjusted effects of the predictor variables on the likelihood of CS delivery. The results are reported as crude odds ratios (OR) and adjusted odds ratios (aOR) with 95% confidence intervals (CIs). A p-value <0.05 was considered statistically significant. Data analysis was conducted using Statistical Product and Service Solutions (SPSS, version 28.0; IBM SPSS Statistics for Windows, Armonk, NY). Listwise deletion was applied to handle missing data; therefore, only cases with complete data across all selected variables were included in the regression model.

## Results

The percentage of CS deliveries in the southern region is 46%. Table 1 presents the background characteristics of the respondents from the southern region of India. The majority of women (60.20%) were in the 25-34 years age group, while only 8.52% were aged 35-49 years. A higher proportion of respondents resided in rural areas (65.40%) compared to urban areas (34.60%). In terms of religion, the majority were Hindu (81.42%), followed by Muslims (13.69%). Regarding employment status, only 26.10% of the women were employed. Education levels among respondents varied, with 57.30% having completed secondary education and 8.90% with no education. Wealth distribution indicated that the majority of respondents belonged to the richest (30.53%), and 5.02% belonged to the poorest quintile. In terms of reproductive history, 45.25% of women had two births, and 4.14% had four or more births. Most respondents (80.93%) had their first birth between 18 and 30 years, 15.73% gave birth before 18 years, and 3.34% had their first birth at or after 30 years. Regarding nutritional status, 53.05% had a normal BMI, and 8.48% were obese. ANC visits showed that 78.29% had at least four visits, and 1.65% did not attend any ANC visits. Regarding childbirth size, 68.35% of the women gave birth to average-sized children, and 24.55% gave birth to large-sized children. The place of delivery varied, with 58.90% delivering in private facilities and 41.10% in public institutions. Finally, 14.40% of women had a history of a terminated pregnancy.

**Table 1:** Background characteristics of the respondents in the southern region

Variables	Frequency	Percentage
Age Group (in Years)		
15-24	6996	31.28
25-34	13,464	60.20
35-49	1905	8.52
Place of Residence		
Urban	7747	34.60
Rural	14,618	65.40
Religion		
Hindu	18,210	81.42
Muslim	3064	13.69
Others	1091	4.89
Respondent’s Working		
No	2523	73.90
Yes	889	26.10
Respondent’s Education Level		
No education	1998	8.90
Primary	1360	6.10
Secondary	12,817	57.30
Higher	6190	27.70
Husband’s Education		
No education	310	9.10
Primary	299	8.80
Secondary	1922	56.50
Higher	873	25.60
Wealth Quintile		
Poorest	1122	5.02
Poorer	3647	16.31
Middle	6428	28.74
Richer	6828	30.53
Richest	4340	19.40
Birth Order		
1	8109	36.26
2	10,120	45.25
3	3209	14.35
≥4	927	4.14
Age at First Birth (Years)		
Less than 18	3518	15.73
18-30	18,099	80.93
≥30	748	3.34
Respondent’s BMI		
Underweight	3558	16.57
Normal weight	11,390	53.05
Overweight	4707	21.90
Obese	1822	8.48
ANC Visits		
0	370	1.65
1-3	4487	20.06
≥4	17,508	78.29
Size of Child		
Large	5477	24.55
Average	15,235	68.35
Small	1599	7.10
Place of Delivery		
Private	12,927	58.90
Public	9017	41.10
Terminated Pregnancy		
No	19,137	85.60
Yes	3228	14.40

Table 2 presents the association between various independent variables and DCS in the southern region of India, analyzed using the chi-square test. The results indicate that age plays a significant role, with higher CS rates among older women. Women residing in urban areas had a higher prevalence of CS deliveries (49.1%) compared to rural women (44.3%), showing a significant association (p<0.05). Religion also plays a role, with Hindu women having a higher percentage of CS (47%) than Muslim women (38.7%). Educational attainment significantly impacts CS rates. Women with higher education had the highest proportion of CS (55.5%), while those with no education had the lowest (30.6%) (p<0.05). Wealth status strongly correlates with DCS, as the proportion of caesarean deliveries increases with socioeconomic status. Women in the richest wealth quintile had the highest CS rates (53.2%), whereas the poorest women had the lowest (25%) (p<0.05). Birth order shows a decreasing trend in CS rates as birth order increases. Women giving birth for the first time had 49.8% of women undergoing CS, while those with four or more births had 21.3% of women (p<0.05). Age at first birth and BMI also demonstrate significant associations, with obesity being linked to the highest CS rates (61.2%). ANC visits significantly influence the mode of delivery, with women who attended more than four ANC visits having higher CS rates (46.5%) (p<0.05). Furthermore, private healthcare facilities recorded significantly higher CS deliveries (63.2%) than public facilities (35.5%) (p<0.05). Variables such as the employment status of the respondent did not show statistically significant associations with DCS.

**Table 2:** Association between independent variables with DCS in the southern region of India DCS: Delivery by caesarean section

Variables	DCS		Chi-square value	p-value
	No	Yes		
Age Group (in Years)	Number (%)	Number (%)		
15-24	4093 (58.5)	2903 (41.5)		
25-34	7044 (52.3)	6420 (47.7)	86.95	0.000
35-49	946 (49.7)	959 (50.3)		
Place of Residence				
Urban	3943 (50.9)	3804 (49.1)	46.72	0.000
Rural	8140 (55.7)	6478 (44.3)		
Religion				
Hindu	9644 (53)	8566 (47)		
Muslim	1879 (61.3)	1185 (38.7)	77.24	0.000
Others	560 (51.3)	531 (48.7)		
Respondent’s Working				
No	1365 (54.1)	1158 (45.9)	1.091	0.296
Yes	499 (56.1)	390 (43.9)		
Respondent’s Education Level				
No education	1386 (69.4)	612 (30.6)		
Primary	863 (63.5)	497 (36.5)	470.50	0.000
Secondary	7078 (55.2)	5739 (44.8)		
Higher	2756 (44.5)	3434 (55.5)		
Wealth Quintile				
Poorest	841 (75)	281 (25)	480.24	0.000
Poorer	2324 (63.7)	1323 (36.3)		
Middle	3498 (54.4)	2930 (45.6)		
Richer	3387 (49.6)	3441 (50.4)		
Richest	2033 (46.8)	2307 (53.2)		
Birth Order				
1	4072 (50.2)	4037 (49.8)	509.91	0.000
2	5153 (50.9)	4967 (49.1)		
3	2128 (66.3)	1081 (33.7)		
≥4	730 (78.7)	197 (21.3)		
Age at First Birth (Years)				
Less than 18	2278 (64.8)	1240 (35.2)		
18-30	9548 (52.8)	8551 (47.2)	291.24	0.000
≥18	257 (34.4)	491 (65.6)		
Respondent’s BMI				
Underweight	2258 (63.5)	1300 (36.5)	433.06	0.000
Normal weight	6473 (56.8)	4917 (43.2)		
Overweight	2202 (46.8)	2505 (53.2)		
Obese	707 (38.8)	1115 (61.2)		
ANC Visits				
0	232 (62.7)	138 (37.3)		
1-3	2477 (55.2)	2010 (44.8)	15.37	0.000
≥4	9374 (53.5)	8134 (46.5)		
Size of Child				
Large	2865 (52.3)	2612 (47.7)	14.09	0.001
Average	8354 (54.8)	6881 (45.2)		
Small	826 (51.7)	773 (48.3)		
Place of Delivery				
Public	8341 (46.5)	4586 (35.5)	2005.27	0.000
Private	3321 (36.8)	5696 (63.2)		
other	421 (100)	0 (0)		
Terminated Pregnancy				
Yes	1537 (47.6)	1691 (52.4)	62.44	0.000
No	10546 (55.1)	8591 (44.9)		
Gender of Child				
Male	6315 (53.6)	5474 (46.4)	2.11	0.145
Female	5768 (54.5)	4808 (45.5)		

Predictors associated with DCS in the southern region of India

Table 3 presents the crude OR and aOR with 95% CIs for factors associated with DCS in the southern region of India. The results highlight several key determinants of CS delivery. Women aged 25-34 years had 1.2 times higher odds of DCS compared to those aged 15-24 years (aOR=1.20; 95% CI: 1.00-1.55), and those aged 35-49 years had 1.5 times higher odds (aOR=1.25; 95% CI: 1.08-1.44). Compared to Muslims, Hindus (aOR=1.64; 95% CI: 1.49-1.79) and women of other religions (aOR=1.43; 95% CI: 1.22-1.67) had significantly higher odds of DCS. Regarding education, women with secondary (aOR=1.18; 95% CI: 1.05-1.33) and higher education (aOR=1.19; 95% CI: 1.04-1.36) had increased odds of DCS compared to those with no education. Wealth status was strongly associated with CS; compared to the poorest, the odds increased progressively from poorer (aOR=1.34; 95% CI: 1.13-1.58) to middle (aOR=1.58; 95% CI: 1.35-1.86) and richer (aOR=1.54; 95% CI: 1.31-1.81) wealth quintiles. Women with lower birth orders had significantly higher odds of DCS: first birth (aOR=2.69; 95% CI: 2.22-3.26), second (aOR=2.74; 95% CI: 2.25-3.24), and third (aOR=1.58; 95% CI: 1.31-1.92) compared to those with birth order ≥4. Age at first birth was positively associated with CS; women giving birth at 18-30 years (aOR=2.69; 95% CI: 2.22-3.26) and ≥30 years (aOR=2.70; 95% CI: 2.25-3.24) had significantly higher odds compared to those who gave birth before age 18. Women with normal weight (aOR=1.15; 95% CI: 1.06-1.25), overweight (aOR=1.55; 95% CI: 1.41-1.71), and obesity (aOR=2.18; 95% CI: 1.92-2.48) had increased odds of CS compared to underweight women. Having one to three ANC visits (aOR=1.45; 95% CI: 1.13-1.84) or ≥4 visits (aOR=1.30; 95% CI: 1.03-1.66) was significantly associated with higher odds of DCS compared to no ANC visits. Women who delivered in private facilities had three times higher odds of DCS compared to those who delivered in public facilities (aOR=3.00; 95% CI: 2.82-3.19). Women who had ever had a terminated pregnancy had higher odds of DCS (aOR=1.21; 95% CI: 1.12-1.32) than those who did not.

**Table 3:** Crude odds ratio (OR), adjusted OR (aOR), and 95% CIs for the variables associated with DCS OR: Crude odds ratio; aOR: Adjusted odds ratio; CI: Confidence interval; DCS: Delivery by caesarean section; Reference: the reference category against which other categories are compared p-value – significance level of association Values <0.05 indicate statistical significance.

Variables	OR (95% CI)	p value	aOR (95% C.I.)	p value
Age Group (in Years)				
15-24	1 (Reference)			
25-34	1.28 (1.21-1.36)	0.000	1.20 (1.00-1.55)	0.000
35-49	1.42 (1.29-1.58)	0.000	1.25 (1.08-1.44)	0.002
Place of Residence				
Urban	1.21 (1.14-1.28)	0.000	0.94 (0.88-1.01)	0.131
Rural	1 (Reference)			
Religion				
Hindu	1.40 (1.30-1.52)	0.000	1.64 (1.49-1.79)	0.000
Muslim	1 (Reference)			
Others	1.50 (1.30-1.72)	0.000	1.43 (1.22-1.67)	0.000
Respondent’s Education Level				
No Education	1 (Reference)			
Primary	1.30 (1.12-1.50)	0.000	1.05 (0.89-1.24)	0.513
Secondary	1.83 (1.65-2.03)	0.000	1.18 (1.05-1.33)	0.005
Higher	2.82 (2.53-3.14)	0.000	1.19 (1.04-1.36)	0.011
Wealth Quintile				
Poorest	1 (Reference)			
Poorer	1.70 (1.46-1.98)	0.000	1.34 (1.13-1.58)	0.000
Middle	2.50 (2.17-2.89)	0.000	1.58 (1.35-1.86)	0.000
Richer	3.04 (2.63-3.50)	0.000	1.54 (1.31-1.81)	0.000
Richest	3.39 (2.93-3.93)	0.000	1.25 (1.05-1.50)	0.012
Birth Order				
1	3.67 (3.12-4.32)	0.000	2.69 (2.22-3.26)	0.000
2	3.57 (3.03-4.20)	0.000	2.74 (2.25-3.24)	0.000
3	1.88 (1.58-2.39)	0.000	1.58 (1.31-1.92)	0.000
≥4	1 (Reference)			
Age at First Birth (Years)				
Less than 18	1 (Reference)			
18-30	1.64 (1.52-1.77)	0.000	2.69 (2.22-3.26)	0.000
≥30	3.50 (2.97-4.14)	0.000	2.70 (2.25-3.24)	0.000
Respondent’s BMI				
Underweight	1 (Reference)			
Normal weight	1.31 (1.22-1.42)	0.000	1.15 (1.06-1.25)	0.001
Overweight	1.67 (1.80-2.16)	0.000	1.55 (1.41-1.71)	0.000
Obesity	2.73 (2.43-3.07)	0.000	2.18 (1.92-2.48)	0.000
ANC Visits				
0	1 (Reference)			
1-3	1.36 (1.09-1.69)	0.000	1.445 (1.13-1.84)	0.003
≥4	1.45 (1.17-1.80)	0.000	1.30 (1.03-1.66)	0.027
Size of Child				
Large	1.02 (0.91-1.14)	0.646	1.06 (0.94-1.20)	0.334
Average	1 (Reference)			
Small	0.90 (0.84-0.96)	0.001	0.90 (0.84-0.97)	0.006
Place of Delivery				
Private	3.12 (2.95-3.29)	0.000	3 (2.82-3.19)	0.000
Public	1 (Reference)			
Terminated Pregnancy				
Yes	1.35 (1.25-1.45)	0.000	1.21 (1.12-1.32)	0.000
No	1 (Reference)			

## Discussion

The prevalence of DCS was 46% in the southern region of India. The WHO recommends an average of no more than 10-15% of births by CS for optimal maternal and neonatal outcomes. The findings of this study showed much higher CS deliveries than the WHO guidelines. Among all other factors, perhaps the place of delivery (private and government) emerged as the most significant factor affecting the prevalence of CS [17]. Despite these factors, some of the rise in the occurrence of CS may be linked to medical conditions or related factors, such as rising obesity and older maternal age at delivery, but the study sample shows a declining prevalence of pregnancy problems, suggesting that the mode of delivery is influenced by non-medical variables. According to the latest research, 21% in India and more than 40% of those in private centers and in the south, DCS was done on women whose pregnancies were deemed low risk [18].

This study shows that, compared to their rural counterparts, women living in urban areas were more likely to give birth by CS. This may be because private health facilities and modern treatment are readily available, and women are more educated and have greater freedom of decision making in urban settings [17,19]. Around the world, the chance of CS increases with maternal age. This study also revealed a significant association between caesarean deliveries and maternal age. Women in the age group 35-49 are more likely to deliver by CS. This is because older women may have a variety of ailments that result in different health issues and force those women to have caesarean deliveries [20]. According to a study conducted in India, women who are overweight have a higher chance of DCS as compared to underweight women [21]. The present study found that secondary and higher educated mothers are more likely to have a DCS than those who are illiterate. Women with higher education are conscious of their health and worry about the safety of their perception of health risks. Furthermore, favoring DCS is due to favorable attitudes about caesarean delivery and the availability of biased information [22]. However, several studies also produced conflicting findings, indicating that women who have less education are more likely to choose CS [23]. Researchers have discovered that higher socioeconomic class is positively associated with CS, giving rise to the rich-poor gap; this conclusion is consistent with other studies [24,25]. In this study, southern states had a high prevalence of DCS, which is consistent with other studies conducted using the NFHS-4 data [26]. The increase in institutional delivery has given rise to a trend towards caesarean birth in all southern states.

This study has several limitations that should be considered when interpreting the findings. Firstly, the reliance on secondary data means the accuracy and completeness of the information are constrained by the limitations inherent in the original survey. Secondly, since some variables are based on self-reported data, recall bias or social desirability bias may influence the reliability of responses, potentially affecting the results. Furthermore, although adjustments for confounding variables were made, there could still be unmeasured confounders that may influence the findings. Finally, the study does not differentiate between medically necessary and elective CS, which is a significant limitation, as the clinical justification for caesarean delivery could influence the interpretation of the findings.

## Conclusions

This study reveals a significant association between CS deliveries and sociodemographic, economic, and healthcare-related factors in the southern region of India. Higher odds of CS were observed among wealthier, more educated women, those with lower birth orders, and deliveries occurring in private healthcare facilities, suggesting potential over-medicalization in the private sector.

These findings highlight the urgent need for context-specific policy interventions to address the rising trend of non-medically indicated CS deliveries. Strengthening public healthcare systems, ensuring strict adherence to evidence-based clinical guidelines, and enhancing awareness among healthcare providers and expectant mothers about appropriate medical indications for CS are essential. Such multi-pronged strategies are crucial for promoting safe, medically justified childbirth practices and safeguarding maternal and neonatal health.
